# Inkjet-Deposited Single-Wall Carbon Nanotube Micropatterns on Stretchable PDMS-Ag Substrate–Electrode Structures for Piezoresistive Strain Sensing

**DOI:** 10.1021/acsami.1c04397

**Published:** 2021-06-02

**Authors:** Henri Ervasti, Topias Järvinen, Olli Pitkänen, Éva Bozó, Johanna Hiitola-Keinänen, Olli-Heikki Huttunen, Jussi Hiltunen, Krisztian Kordas

**Affiliations:** † Microelectronics Research Unit, University of Oulu, Erkki Koiso-Kanttilan Katu 3, FIN-90570 Oulu, Finland; ‡ VTT Technical Research Centre of Finland, Kaitoväylä 1, FIN-90590 Oulu, Finland

**Keywords:** printed electronics, stretchable materials and devices, piezoresistive sensing, strain, pressure, and bending sensors

## Abstract

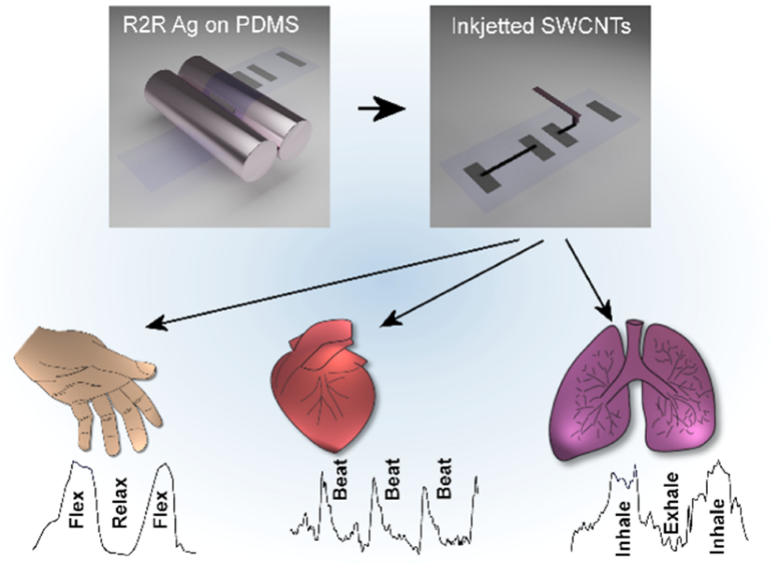

Printed piezoresistive strain sensors based on stretchable roll-to-roll screen-printed silver electrodes on polydimethylsiloxane substrates and inkjet-deposited single-wall carbon nanotube micropatterns are demonstrated in this work. With the optimization of surface wetting and inkjet printing parameters, well-defined microscopic line patterns of the nanotubes with a sheet resistance of <100 Ω/□ could be deposited between stretchable Ag electrodes on the plasma-treated substrate. The developed stretchable devices are highly sensitive to tensile strain with a gauge factor of up to 400 and a pressure sensitivity of ∼0.09 Pa^–1^, respond to bending down to a radius of 1.5 mm, and are suitable for mounting on the skin to monitor and resolve various movements of the human body such as cardiac cycle, breathing, and finger flexing. This study indicates that inkjet deposition of nanomaterials can complement well other printing technologies to produce flexible and stretchable devices in a versatile manner.

## 1 Introduction

Stretchable electrical components and devices play a pivotal role in future smart applications implemented in apparel, sports, and medical equipment, as well as in various tactile systems. The typical functions include (i) simple electrical wiring and interconnections for sensors, displays, and other devices,^1−3^ (ii) sensing of mechanical deformations, stresses, and forces, which then enable collection of data on human motion and vitality parameters or can provide information for feedback loops in robotic control,^4^ and (iii) electrodes and collectors for energy scavenging and storage.^5,6^ Woven-type textiles^7^ based on yarns of composites^8^ or core–shell assemblies^9^ of conductive nanomaterials and polymers offer structural stretchability similar to those of Kirigami patterns^10−12^ without the need of intrinsic elasticity of the materials involved. On the other hand, intrinsically stretchable materials are composites of elastomers (silicones,^13^ hydrogels,^14^ and rubber-like materials^15^) and percolated three-dimensional networks of conductive fillers [metal nanowires and nanoflakes,^16,17^ carbon nanotubes (CNTs),^18^ and graphene] or two-dimensional films of interconnected conductive nanomaterials applied on the surface of the elastomer.^19,20^

When it comes to practical applications of such stretchable electrical components, a large number of devices are required at a low cost; therefore, it is important to explore technologies that support mass production and/or are compatible with other enabling technologies of the corresponding industry. In this context, we have developed new methods to print not only the stretchable electrically conductive micropatterns but also the substrate using roll-to-roll (R2R) printing. In our approach, we applied a flexible but nonstretchable support film (commercial aluminized paper substrate) upon which polydimethylsiloxane (PDMS) was dispensed and spread R2R, making it suitable for in situ embossing, thus forming microfluidic channels after curing the polymer film.^21^ In another approach, we used the already cured PDMS film on the polyethylene terephthalate (PET) carrier as a substrate for continuous rotary screen printing of a silicone-based Ag paste.^22^ After curing the paste, the printed conductive patterns were flexible and stretchable and were feasible for bonding discrete surface mount components, and the as-made film could be laminated with another layer of PDMS. The abovementioned methods proved to be robust and versatile, considering that the running speed of the films in the printing machine (up to 2 m·min^–1^) allows for truly large-scale production of stretchable components.

In our current work, we elaborate further on the technology and combine the printed stretchable PDMS-Ag substrate-electrode films with inkjet-printed single-wall CNT (SWCNT) micropatterns^23−25^ to produce strain sensors that are ubiquitous components, for example, in soft robotics, for high precision grippers and tactile systems;^26,27^ in medical and sports equipment, for measuring blood pressure and monitoring movement/flexing body parts;^28,29^ in civil engineering, to analyze deformations and displacements in building infrastructure;^30^ and they may find good use in automotive,^31^ aeronautics,^32^ and other applications as well. Here, we show that under optimized conditions, highly conductive microscopic line patterns of SWCNTs with sheet resistance below 100 Ω/□ deposited by simple inkjet printing between stretchable Ag electrodes on PDMS are feasible for sensitive monitoring of mechanical strain with a gauge factor of up to 400 and a pressure sensitivity of ∼0.09 Pa^–1^. Apart from tensile deformations, the sensors respond to flexing and are feasible to monitor heartbeats at the radial artery.^33^

## 2 Experimental Section

**Figure 1:**
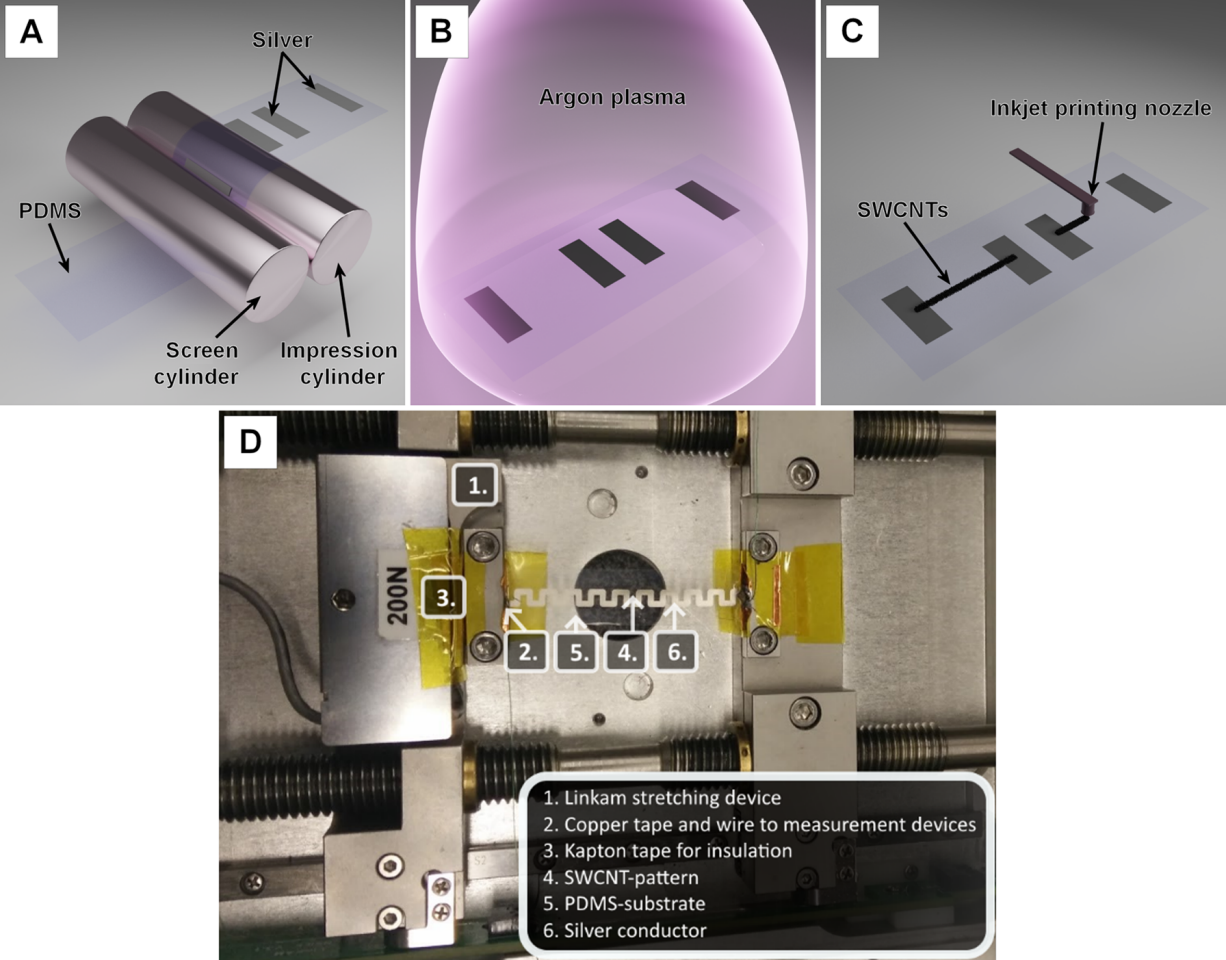
Illustration of the subsequent process steps of sensor preparation: (A) screen printing of the silver contact pads, (B) argon plasma treatment, and (C) inkjet printing of the SWCNT patterns. (D) Top view of the experimental setup to measure the piezoresistive behavior of the printed SWCNT micropatterns [panel (D) is reproduced from ref (33) Ervasti, H. Inkjet-Printed SWCNT Conductors and Sensors on PDMS. M.Sc. Thesis, University of Oulu, 2020].

Stretchable PDMS-Ag substrates were made as described in our previous work.^22^ The Ag paste (Creative Materials 125-19FS) was screen-printed (rotary screen Gallus GV steel mesh 200 in.^–1^) on the PDMS substrate (100 μm thick PDMS on the PET carrier, ELASTOSIL Film 2030, Wacker-Kemi) with a speed of 2 m·min^–1^ and then cured at 140 °C in a convection air oven. Depending on the number of prints, the thickness of the Ag electrodes is about 7 μm for a single layer and 15 μm for two overlapping layers with corresponding sheet resistances of 0.27 and 0.15 Ω/□. For our experiments, the substrate was cut into pieces of 10 mm × 25–50 mm size. The Ag paste containing the silicone elastomer binder adheres well to the PDMS substrate, withstands continuous stretching cycles reasonably well,^22^ and is not affected negatively by the postprocessing steps such as Ar plasma treatment or inkjet printing (Figure 1A–C).

Ar plasma treatment was carried out using an Oxford PlasmaLab Plus facility (200 W, 20 sccm Ar flow at 20 mTorr). The samples analyzed using atomic force microscopy (AFM) (Bruker Multimode AFM with the μmasch NSC18 probe) were treated for 10 min, whereas the ones used in the printing experiments were etched for 13 min.^32^

The ink is expected to meet several material property-related parameters to be able to jet through the nozzles of the piezoelectric injection transducer, including viscosity, surface tension, particle size, and solvent drying time. To meet the process window of the printer, we apply a 1:1 mixture (by weight) of deionized water and dimethylformamide as solvent. Also, pristine CNTs, which are highly hydrophobic, are nearly impossible to disperse in water, and thus, carboxylic acid-functionalized single-wall CNTs (SWCNT-COOH) (Sigma-Aldrich 652490) were used. Their polar side groups can interact with polar solvents, thus stabilizing the solid particles in the medium. Solid drying particles such as surfactants (e.g., sodium dodecyl sulfate), although they could have been beneficial in terms of surface tension and possibly deter early CNT agglomeration, were avoided so that the finished product would have the least number of contaminants and particles to affect conductivity and other properties. To minimize the challenge of nanotube agglomeration, we first thoroughly dispersed the nanotubes in the solvent for 2 min in a magnetic mixer, which was then followed by ultrasonic dispersion (Finnsonic m12 200w/800w) for 30–60 min at 50 °C. The obtained dispersion was then centrifuged (Hettich Zentrifugen universal 320) at 3500 rpm for 10 min, and then, the supernatant was collected. After repeating the centrifugation and collection steps 4 times, a very stable SWCNT ink with a concentration of 0.316 ± 0.017 mg/mL was obtained.^33^

The inkjet-printed patterns were printed using a Dimatix DMP-2850 materials printer. Jetting velocities were adjusted to 4–7 m/s, while either drop spacing or the amount of deposited CNT was varied by layering. The temperature of the printing nozzle and sample holder stage was set at 23 and 60 °C, respectively.

The microstructure of the substrates and inkjet-deposited SWCNT patterns was analyzed by scanning electron microscopy (SEM, Zeiss ultra plus). The samples were sputtered up with Pt of 1.4 nm size and grounded using carbon tape to deter surface charging.^33^

The contact angles (advancing edge) of water and the SWCNT ink were measured by analyzing digital images (ImageJ) of the droplets dispensed on the substrates taken using a Canon Powershot SX10 IS (side view, at a distance of 5–6 cm).^33^

For electrical measurements, a Wentworth probe station was used to make contacts on the Ag pads of the substrate. The contact resistance and other series resistances combined were negligible compared to the resistance of the printed SWCNT patterns. The sheet resistance of the SWCNT line patterns was calculated from measured resistances considering a mean track width of 46.3 ± 5.8 μm and length of 1000 μm.^33^

*I*–*V* measurements were carried out using a Keithley 2636A with an automated LabVIEW script (−2 to +2 V), with a current limit of 1 mA, hysteresis enabled, 0.1 V voltage step, and 100 ms delay in between the samples.^33^

For the stretching tests, we used a Linkam TST 350E stretch stage with a Linkam T95-PE controller (positional resolution of 10 μm). The support from the back-side was removed, and the samples were fixed to the stretch stage with a copper tape facing to the silver to give a good and reliable contact (Figure 1D). The results were compared with a reference sample containing no SWCNTs to confirm that the nanotube network is the source of the piezoresistive effect, and the Ag electrodes have an insignificant contribution to the variation of resistance.^33^

For the real-time resistance measurement, Keithley 2636A with an automated Labview script was used. The setup was confirmed to give accurate resistance and current/voltage measurements up to 43 samples/s, as 23 ms is the minimum step delay in the resistance range of 20–300 kΩ. The two measurement devices were scripted to start and end at the same time, and the results were afterward scaled to the same time domain. The gauge factor was later derived from the strain curves in Origin Pro 2019b.^33^

In the radial artery pulse test, the sensor was put over the radial artery and pressed manually with a finger to find the correct spot and pressure. It was then connected in series with a 22.5 kΩ wire wound resistor, and a voltage of 8.74 V was sourced from an Agilent E3614A device, whereas the temporal variation of potential drop on the sensor was measured using an Agilent DSO-X 3024A oscilloscope. Lastly, the signal was post-processed with a low pass 25 Hz FFT filter (Origin Pro 2019) to remove the 50 Hz mains hum from the data.^33^

For the finger flexing and chest movement upon breathing tests, the ends of the sensor membrane were fixed on the skin by copper tape. For monitoring finger flexing, the sensor was placed longitudinally between the second and third joints of an index finger. For monitoring breathing, the sensor was placed almost vertically to the upper part of the right chest. The ends of the copper tape were connected using a thin wire to a Keithley 2636A sourcemeter. The sourcemeter is controlled by an automated Labview script to source a small measurement voltage into the sensor every 100 ms and measure the resulting current from which the resistance can finally be calculated.

The temperature dependence of resistance of the line-shaped printed SWCNT pattern was measured in a Linkam THMS600 heating stage with a computer-controlled Hewlett–Packard 3458A multimeter applying 1 V bias in 200 sccm synthetic air flow (80% N_2_, 20% O_2_). For the analysis of humidity response, the inserted air was humidified by bubbling it through deionized water and measured with a commercial Adafruit DHT22 sensor.

## 3 Results and Discussion

### 3.1 Surface Wetting of the Substrate

Inkjet printing of well-defined microscopic patterns of nanomaterials on pristine PDMS is quite cumbersome due to the extremely poor wetting of the surface with inks and solvents. Ink droplets do not spread on the surface; once they become dry, their solid content leaves irregular stains, which attract and merge with subsequent droplets jetted in their proximity, making it more than challenging to create any microscopic patterns with good line definition. However, the small surface energy of most technical polymers can be increased by, for example, alkali or acid treatment and plasma etching. Here, we selected argon-plasma bombardment that we successfully implemented earlier to improve the wettability of PET films.^34^ Plasma treatment times were varied between 2 and 30 min. As was found, even short plasma etching makes PDMS hydrophilic; however, the surface relaxes quickly, leaving very limited time for subsequent printing. On the other hand, prolonged treatments resulted in nonuniform hydrophilic surfaces, especially around the printed silver conductive paths (probably due to the partial sputtering of Ag electrodes). The best results were achieved with 13 min of treatment and 5–10 h of waiting afterward.

**Figure 2:**
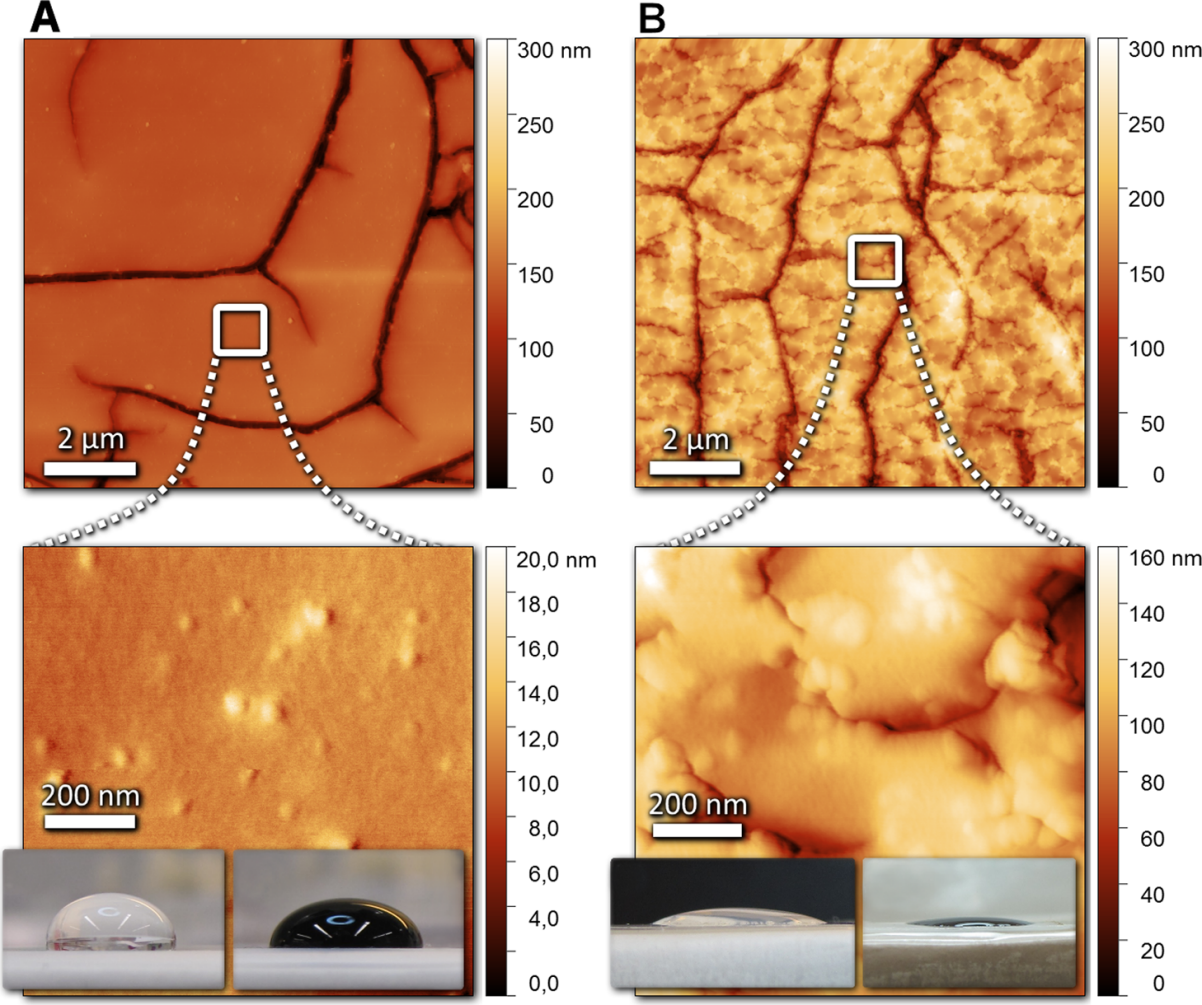
AFM topology scans of 10 × 10 and 1 × 1 μm^2^ areas of (A) pristine and (B) Ar plasma-treated PDMS surfaces (10 min, 200 W). Insets show water and SWCNT ink droplets deposited on the corresponding surfaces [the figure is reproduced from ref (33) Ervasti, H. Inkjet-Printed SWCNT Conductors and Sensors on PDMS. M.Sc. Thesis, University of Oulu, 2020].

AFM topology scans show that the pristine PDMS surface is smooth but decorated with some fingerprint-like narrow grooves of 200 nm depth. After plasma treatment, the surface structure becomes corrugated with more grooves and bulging nodules of 100 nm height. The high surface roughness is also visible with naked eye due to surface scattering of light (not shown here). Contact angle measurements show great fluid spread on the treated surface (having 30 and 15° for water and ink, respectively) in reference to the original PDMS (with 95 and 85°). It is worth noticing that the angles are systematically lower for the used SWCNT ink because of its lower surface tension in reference to water (Figure 2).

**Figure 3:**
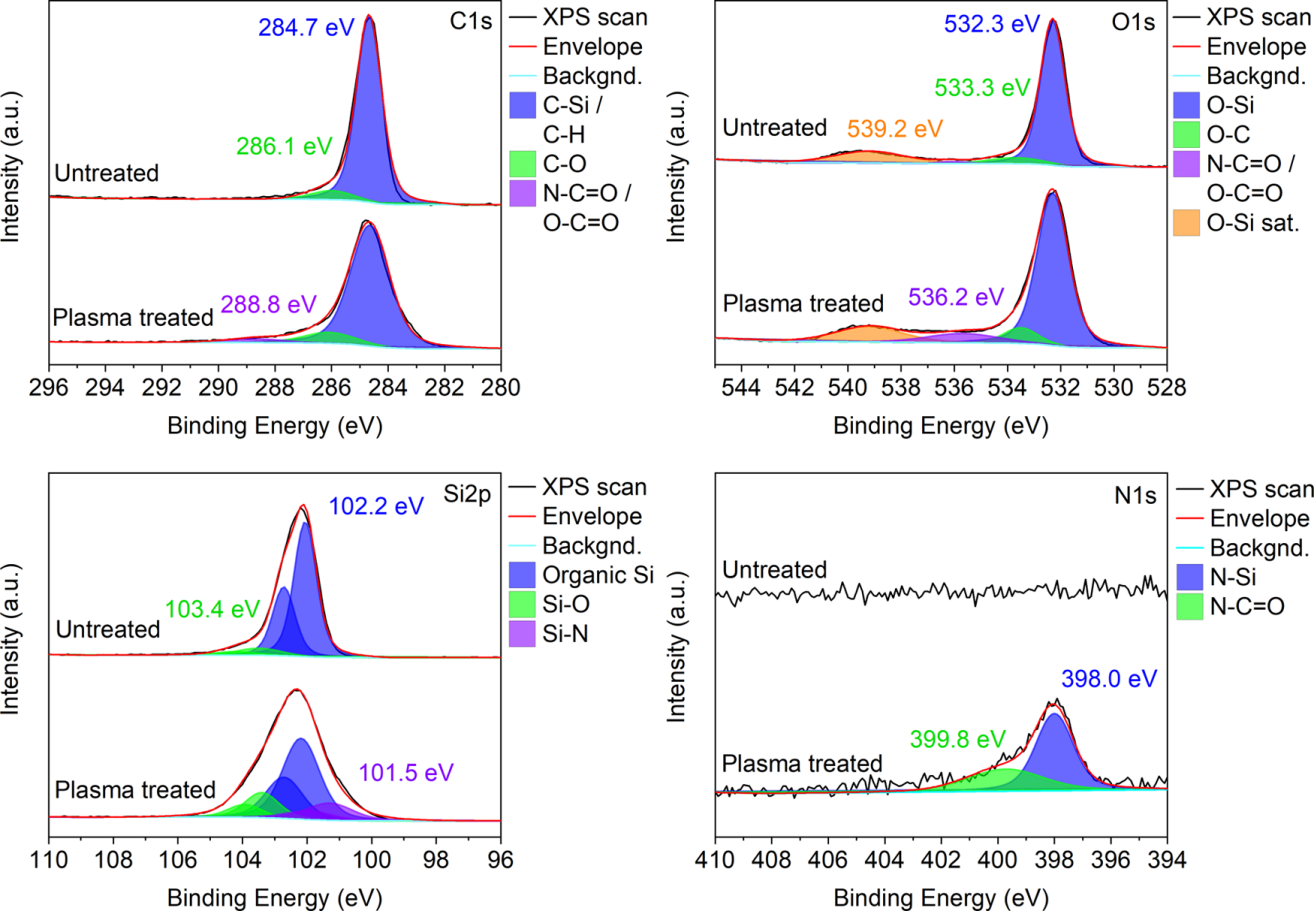
Resolved X-ray photoelectron spectra of C 1s, O 1s, Si 2p, and N 1s peaks before and after Ar plasma treatment of PDMS at 200 W for 13 min.

X-ray photoelectron microscopy is used to investigate the effect of the argon plasma treatment on the surface chemistry of PDMS substrates (Figure 3). In the resolved C 1s, O 1s, and Si 2p regions, new O bonds appear after the plasma treatment. The strongest increase is visible in the Si 2p region where the increase of peak intensity of the Si–O bond at 103.4 eV indicates the formation of silanol groups on the PDMS surface, thus increasing its hydrophilicity. Besides the Si–O bond, the formation of Si–N bonds (observed at 101.5 eV) also contributes to the change of the surface energy. The presence of the N–Si bonds is also seen in the N 1s spectrum (at 398.0 eV) along with a small amount of N–C=O (at 399.8 eV), which can also be traced in C 1s (at 288.8 eV) and O 1s (at 536.2 eV) spectra.^35^ Overall, ∼5 at. % increase of surface oxygen (and ∼1 at. % nitrogen) after the plasma treatment is observed by X-ray photoelectron spectroscopy, which is slightly lower compared to previous studies^36−38^ likely due to lower plasma treatment power and exposure time used in our study. It is worth noting that a small amount of ∼0.5 at. % of entrapped argon in the polymer matrix was detected in the plasma-treated sample (Supporting Information, Figure S1).

### 3.2 Optimization of Inkjet Printing and Electrical Properties

Carboxyl-functionalized SWCNTs dispersed in the water–DMF 50 wt % mixture was used as the printing ink (concentration of 0.316 ± 0.017 mg/mL and pH ∼ 6) in the experiments. *N*,*N*-Dimethylformamide (DMF) provides good nanotube dispersibility, and its low vapor pressure helps to avoid rapid drying of the ink in the printing nozzles and maintains the necessary viscosity important for printability.

**Figure 4:**
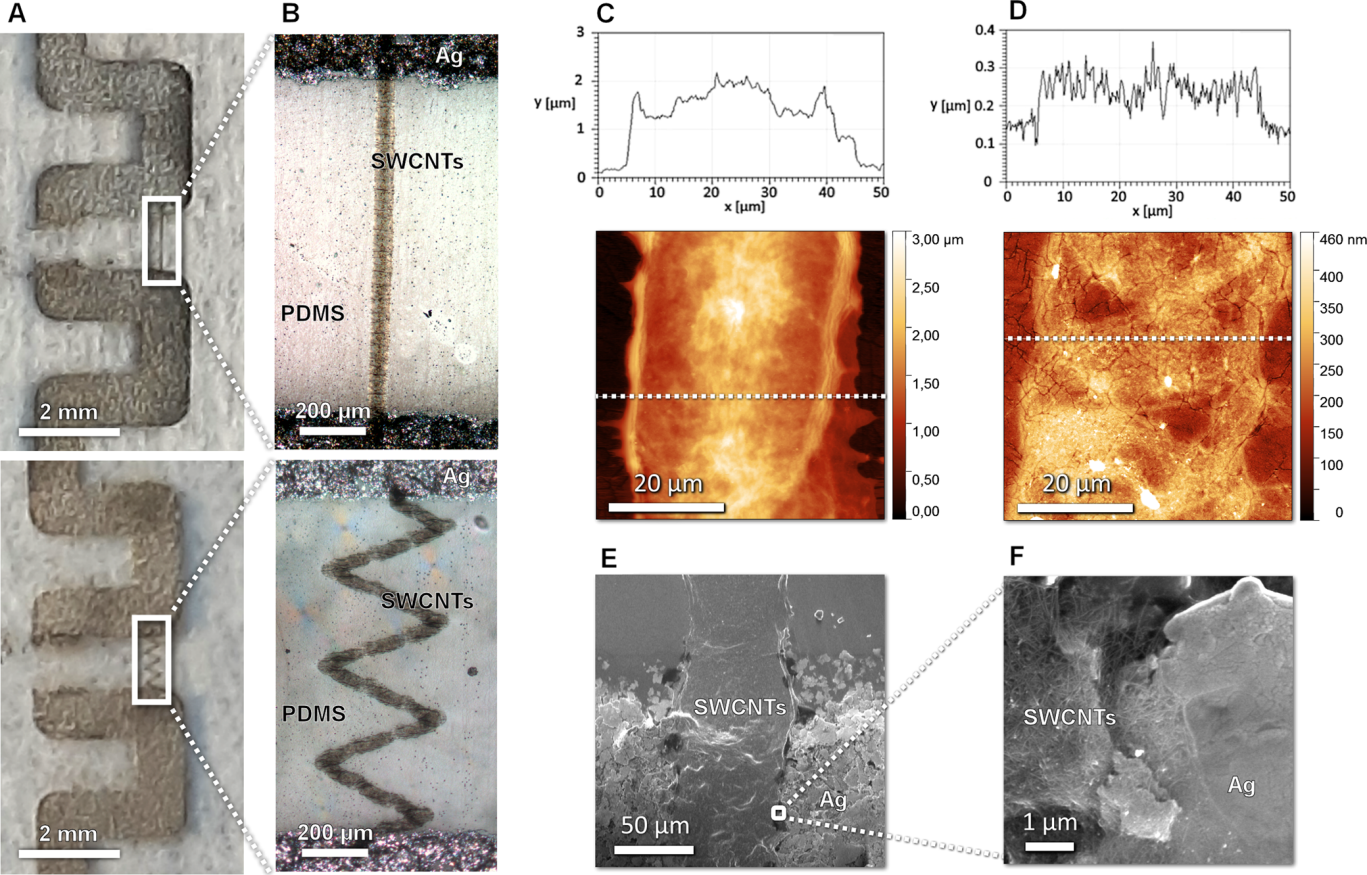
(A,B) Optical images of the inkjet-printed SWCNT straight line and zigzag micropatterns on the PDMS-Ag substrate having 1 mm gaps between the electrode pads. (C,D) AFM topology scans showing ∼1.5 μm and 130 nm thickness profiles for 12-layer and single-layer patterns, respectively. (E,F) SEM images with low and high magnification taken from the step edge of the Ag electrode showing that the printed CNT pattern is conformal, that is, follows the surface topology of the surface [panels (C–F) are reproduced from ref (33) Ervasti, H. Inkjet-Printed SWCNT Conductors and Sensors on PDMS. M.Sc. Thesis, University of Oulu, 2020].

To explore the dependence of topology and electrical resistance on the printing parameters, we varied drop spacing and layer count (the number of repeated printing scans over the same pattern) and analyzed the effect of the order of droplet ejection (i.e., first vs subsequent droplets). The line patterns of the nanotubes with a resolution of ∼50 μm and good edge definition could be deposited on the plasma-treated surface between the Ag electrodes using even a single print scan. The nanotubes seamlessly cover the surface and also the step at the edge of the Ag electrode [i.e., the line pattern of the printed SWCNTs is conformal (Figure 4)].

**Figure 5:**
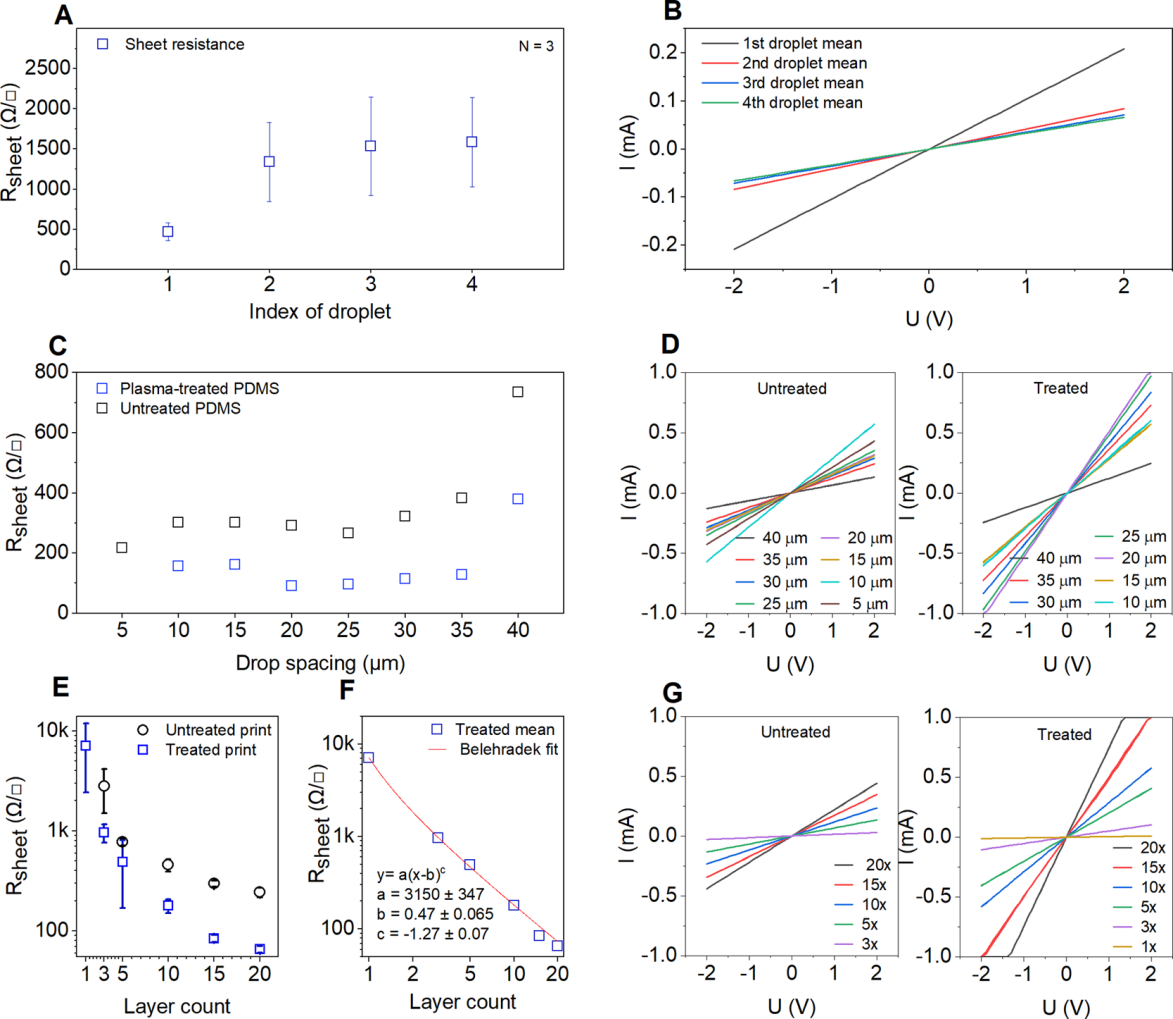
(A) Idling effect of the printing nozzle on sheet resistance of the deposited pattern with the first ink droplet having higher nanotube concentration than the subsequent ones and (B) corresponding *I*–*V* curves. (C) Effect of drop spacing on sheet resistance and (D) corresponding *I*–*V* characteristics measured for line patterns on untreated and treated PDMS substrates. (E) Sheet resistance as a function of the repeated print layer number and (F) Belehradek power function fit for the patterns deposited on plasma-treated PDMS. The inset shows the fitting parameters. (G) *I*–*V* curves measured for the corresponding data points shown in panel (E) [the figure is reproduced from ref (33) Ervasti, H. Inkjet-Printed SWCNT Conductors and Sensors on PDMS. M.Sc. Thesis, University of Oulu, 2020].

Because of the relatively slow drying and irregular merging of the jetted SWCNT ink droplets on the surface, it was cumbersome to print horizontal lines along the scan direction of the printing head. Therefore, we chose a different strategy and printed several parallel vertical lines (i.e., perpendicular to the print head scan direction), adding individual droplets to each line in a particular scan. As was observed, the very first droplets in the horizontal scan always produce a darker deposit than the subsequent ones, making the leftmost vertical line more conductive than the others (Figure 5A,B). Because the droplets ejected by the printer have the same nominal volume (10 pL, provided the nozzles are clean), the reason for the first droplet effect in a horizontal scan (i.e., to consistently produce a more conductive line) is attributed to a more concentrated ink. The reason for having more concentrated ink in the first droplet is likely due to the evaporation of solvent at the air–ink interface, when the printhead is moving but not ejecting (while the designed meniscus vibration mixes the more concentrated ink surface back into the suspension to prevent a complete blockage). As the horizontal scan progresses, the delay time between adjacent droplet shooting is small; thus drying does not affect the ink concentration anymore, and subsequent droplets rapidly reach the concentration of the original inks.

Because of the higher amount of SWCNTs deposited within the first droplet, in further experiments, we used it to print the conductive patterns. Variation in droplet volumes, ejection velocities and angles, and various drying effects take place on the surface, which all together introduce uncertainties in the properties of the printouts. Such deviations most likely could be reduced with the use of more robust industrial equipment; although in the sensor applications we show in our report, some sample-to-sample variation of resistance is not detrimental.

The effect of drop spacing on the electrical properties was assessed by varying this parameter between 5 and 40 μm while keeping the amount of deposited SWCNTs nearly constant (803–827 droplets applied in each pattern). As shown in Figure 5C,D, drop spacing has negligible influence, as long as it is smaller than the diameter of the deposited droplet on the surface (∼40 μm). It is worth mentioning that the adjacent droplets of 5 μm drop spacing were found merging irregularly due to imperfect drying of the previous droplets; thus, in the subsequent experiments, we applied 20 μm drop spacing.

The conductance of the line patterns shows a strong dependence on the amount of deposited SWCNTs adjusted by repeating inkjet scans over the same pattern (layer count from 1 up to 20). Already a single layer of SWCNT is reasonably conductive with a sheet resistance of 7 kΩ/□, which significantly decreases as more layers are added reaching values below 100 Ω/□ at 15 and 20 layer counts (Figure 5E) comparable to other reported data for printed CNTs on flexible substrates such as cloth fabrics^39^ and paper.^40^ The dependence follows a power function *y* = *a*(*x* – *b*)^*c*^ of percolating networks and, according to the fitting parameters, have a percolation threshold *b* ∼ 0.5 (i.e., percolation is achieved already with the first printed layer) and power exponent *c* ∼ 1.3 (Figure 5F), where the latter is in excellent agreement with the expected value for 2D networks (1.33).^41^ Because of the well-percolated networks in our experiments, several percolation paths of entirely metallic nanotubes exist in the printed patterns, and thus, their *I*–*V* curves are linear implying ohmic electrical transport (Figure 5B,D,G).

### 3.3 Piezoresistive Properties

In CNT-based strain sensors, the resistance change is mainly caused by the deformation of the CNT percolation network due to disconnections and reconnections of the CNT junctions during strain.^42^ In a simple picture, under tensile strain, the network of nanotubes becomes sparse and a reduced conductance is observed similar to any other piezoresistive materials based on networks of nano- or microparticles. On the other hand, the mechanism is a bit more complex when we consider the macroscopic geometry of the conductive patterns deposited on the substrate. In the case of straight line patterns, the stress (and also the strain) is evenly distributed along the structure, and the local strain is the same as the macroscopic strain of the device. In contrast, when introducing curves in the structure, the length of the pattern increases; thus, the local strain is reduced (viz. the structure works like a spring) and the strain sensitivity of such sensors is decreased (while the maximum applicable strain is increased). Importantly, the shape and the curvature of the pattern also influences the local strain. Any irregularities or abrupt variations of the pattern geometry have high stress concentrations^43^ in the locations with a small radius of curvature, resulting in high local strain response such as those present in zigzag-patterns (having sharp rectangular corners). In comparison, structures with smooth envelopes such as those of horseshoe, meander, and sinusoidal patterns have smaller stress concentrations.^42,44^ Accordingly, after the straight lines, zigzag patterns provide the highest gauge factors, followed by square wave-shaped patterns, whereas patterns with rounded turns (sinusoidal, meander, or horseshoe) have been reported to have the lowest sensitivities and gauge factors due to the more evenly distributed strain.^44,45^ Therefore, straight line-based patterns have been widely used in commercial strain sensors as these enable the highest sensitivities and gauge factors. In contrast, curved periodic geometries (sinusoidal or meander) are preferred when the functionality of the printed pattern is meant to be an interconnection with high durability and the lowest possible sensitivity to strain.^45^

**Figure 6:**
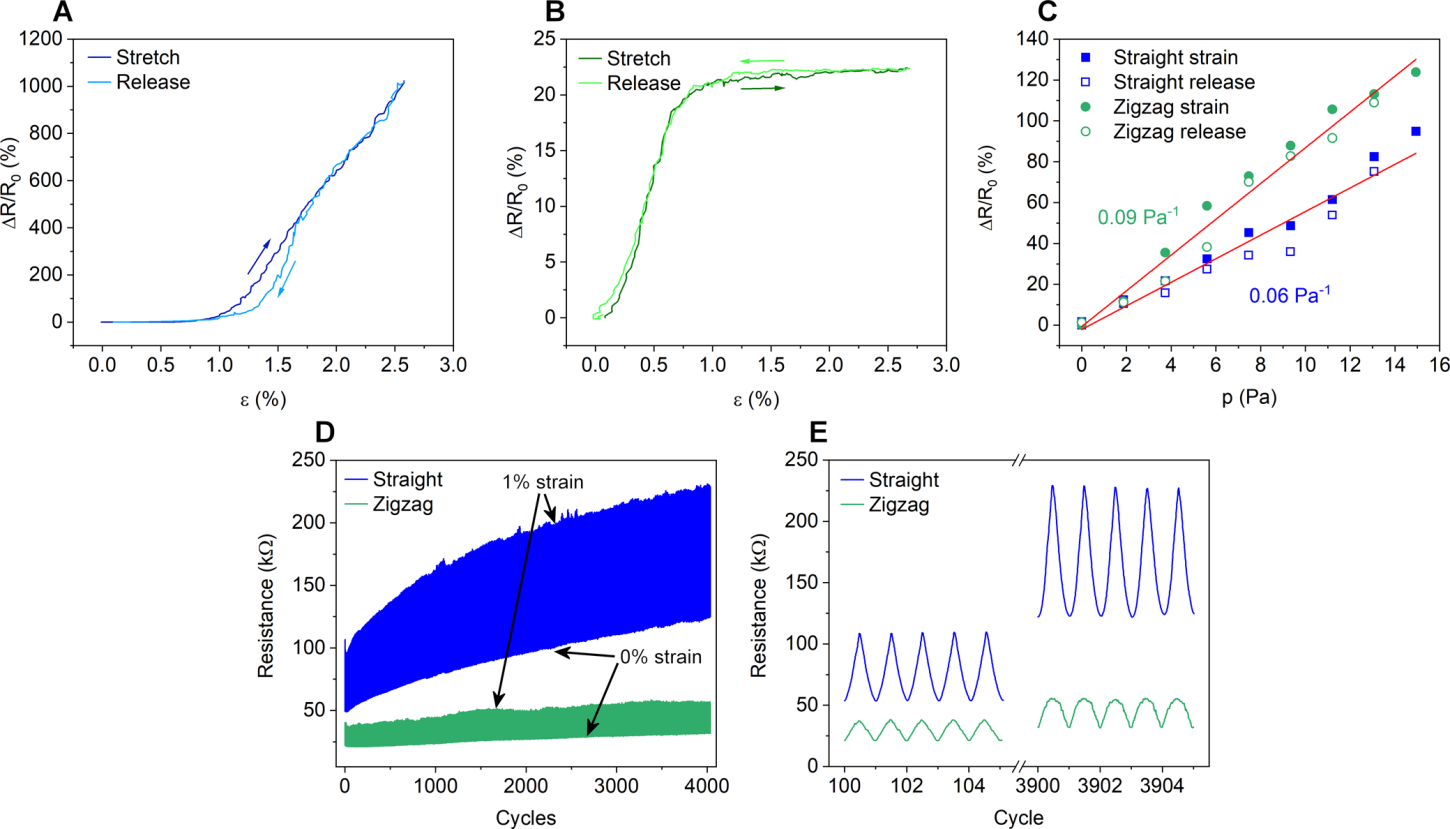
Relative change of the resistance of a printed piezoresistive sensor device with (A) straight line and (B) zigzag patterns upon tensile strain. (C) Sensor responses to pressure and linear fitting with their corresponding pressure sensitivity values. (D) Resistance change during 4000 cycles under 1% stain and (E) close-up of the resistance signal during cycles 100 to 104 and 3900 to 3904.

The piezoresistive behavior of two different micropatterns (straight line and zigzag) was studied using mechanical tensile testing under strains up to 2.5% (Figure 1D). When strained, the resistance of the patterns increases ∼10-fold with the straight line pattern (Figure 6A) but only ∼20% with the zigzag pattern (Figure 6B) where the tensile strain is almost perpendicular to the SWCNT line direction in the pattern, demonstrating how the response characteristics can be greatly modified by the printed pattern design. The sensor responses are reversible and have low hysteresis with low stage cyclic rates (50 mHz, ε_max_ = 2.5%). With higher deformation rates and applied maximum strains (*f* = 200 mHz, ε_max_ = 3.5%), the response shows hysteresis in the stretch–release cycle (Supporting Information Figure S2), which is likely to be caused by the dynamics of deformations in the local microstructure of CNTs in the tangled networks.^46^ Our hypothesis on the strain dependence of the sensors dynamic response (and hysteresis) is actually supported well by the study of Michelis et al.,^47^ showing that CNTs printed on ethylene tetrafluoroethylene respond within ∼4 and ∼20 s, when the applied strains are 500 and 1500 με, respectively. The gauge factor of the straight line pattern is in the same range as zigzag in low (<1%) strains, after which, it increases more steeply, whereas in the zigzag pattern, the calculated gauge factor begins to drop after 0.65% strain. The calculated maximum gauge factors of the devices are 400 at 2.5% strain and 28 at 0.65% strain for straight line and zigzag patterns, respectively, which are well comparable with previously reported results on piezoresistive sensors based on nanosized carbon such as CNTs,^47−51^ graphene,^52−57^ reduced graphene oxide,^58−60^ or graphitic carbon^49,61^ (Table S1). It is important to note here that the series resistance of the Ag electrodes is negligible compared to the resistance of the SWCNT pattern and thus has an insignificant effect on the overall piezoresistive behavior of the sensors.

To analyze the pressure sensitivity of the printed nanotube networks, the device was placed horizontally, clamped from its ends (similar to that in Figure 1D) to form a suspended membrane, and then loaded from the top using discrete weights, thus straining the sensor structure. Loads up to 0.6 g corresponding to ∼15 Pa pressure were applied (considering a total affected area of ∼4 cm^2^). The measured relative changes of device resistances are linear within the applied pressure range and show only insignificant hysteresis during the stress-release cycle. From the slopes of the linear fits, the pressure sensitivities of devices are 0.06 and 0.09 Pa^–1^ for straight line and zigzag patterns, respectively (Figure 6C). Such pressure sensitivity values are comparable to those reported for the state-of-the-art aerogel composites of CNTs, reduced graphene oxide, and carbon nanofiber-based devices.^62^ We note here that the pressure sensitivity might be improved even further by applying thinner PDMS substrates than the ones used in the current experiments (∼100 μm) because inherently smaller stress would be required to result in the same strain of the substrate and the SWCNT micropattern.

To investigate the reliability performance, the printed sensors were subjected to 4000 stress-release cycles with 1% strain (Figure 6D). After 4000 cycles, both straight line and zigzag sensor patterns had an increase in their base resistance with ∼50 and ∼120%, respectively. This is typical for open CNT films without encapsulation due to the accumulation of small irreversible rearrangements of CNTs occurring during the strain-release cycling.^63^ The smaller resistance change in the zigzag pattern can be explained by smaller local strains as the total pattern length is larger over the same sensor length. Encapsulation (e.g., by lamination or overcoating) of the device would likely further improve the retention performance.^22^ On the other hand, the gauge factors of the two different patterns based on the data shown in Figure 6D remained nearly constant; thus, we opted out encapsulation to avoid adding one more tedious process step in making the sensors.

**Figure 7:**
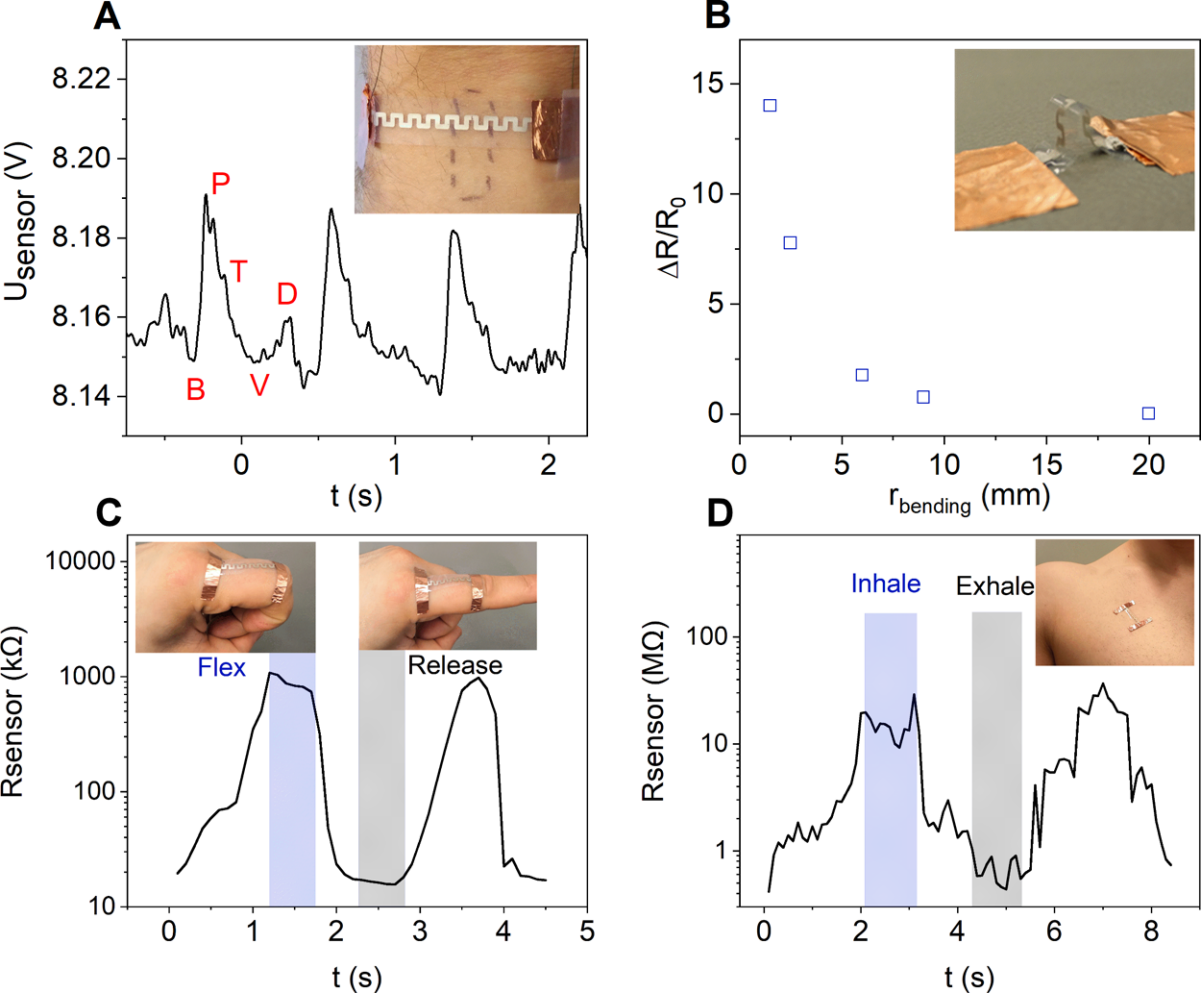
(A) Pulse measurement on the radial artery with the printed sensor with resolved pulse features including B: base point, P: percussion wave, T: tidal wave, V: valley, and D: dicrotic wave. (B) Relative change of the resistance (*R*_0_ = 80 kΩ) as a function of the surface curvature (convex, i.e., the SWCNTs located on the top of the structure are under tensile stress). (C) Sensor response on monitoring finger flexing and (D) chest movement upon breathing. A straight line pattern was used in the measurements [panels (A,B) are reproduced from ref (33) Ervasti, H. Inkjet-Printed SWCNT Conductors and Sensors on PDMS. M.Sc. Thesis, University of Oulu, 2020].

There has been an increasing interest in developing simple, low-cost, and multifunctional smart skin adhesive patches for medical applications.^64^ As Young’s modulus of the used PDMS substrate (Elastosil 2030, ∼1.2 MPa)^65^ is close to that of the human skin on the forearm (depending on the age and the measurement method: 0.42,^66^ 1.0,^67^ and 0.08–0.26 MPa^68^), it is anticipated that the sensor mounted on the skin is capable of detecting small pressure variations caused by arterial pulses. To remove noise in our experiments, which originated mostly from 50 Hz mains hum, the recorded data were post-processed with a low pass 25 Hz fast Fourier transform filter (Origin 2019b). As shown in Figure 7A, the sensor is sufficiently sensitive to resolve the pressure waves in the pulses. We assume that the sensing mechanism is based on the change of the curvature of the structure caused by the pressure fronts that deform the sensor and thus strain the printed SWCNT network on the surface. To verify this hypothesis, the response of the sensor to flexing is elaborated further by bending a sensor around cylindrical objects to create a convex curvature while measuring the change of its electrical resistance. The results indicate that bending increases the resistance as expected (Figure 7B). Bending radii below ∼10 mm could be detected, and the sensor was found to maintain its functionality even at a 1.5 mm radius. Furthermore, the sensor responds well when its ends are given a fixed location on the human skin and the membrane is allowed to stretch together with the movements of the body part. This way, finger joint movement (Figure 7C) and breathing (Figure 7D) were monitored with an excellent resolution.

Considering practical applications in wearable electronics, we need to consider the dependence of sensor response to temperature and humidity. Accordingly, we conducted experiments within the parameter windows from 25 to 100 °C and 10 to 80% (Figure S3). The resistance of the printed CNT network was found to decrease with increasing temperature by 8% (from 25 to 100 °C), as reported earlier, with thick inkjet-printed SWCNT films^25^ due to the temperature dependence of electron hopping and transport through Schottky junctions between the nanotube contacts in the CNT film.^25,70^ Humidity, on the other hand, increased the resistance up to 15% in the relative humidity range of 10–65%. This is due to the p-type semiconducting nature of SWCNTs (viz. in the network, one-third of the nanotubes is metallic and the rest is semiconducting) and water molecules that adsorb on the surface and act as weak reductants (electron donors), by which holes in the SWCNTs are localized, thus decreasing the conductivity.^25^ On the other hand, as the humidity increased further (from 65 to 80%), the resistance did not increase any further (actually the trend turns the opposite), which is likely due to the densification of the nanotubes caused by capillary forces^69^ of condensing water that can induce the formation of nanotube bundles in the network. We must note here that the influence of both temperature and humidity on the device resistance is much smaller (even in extreme environmental scenarios) than the piezoresistive effect. Anyhow, if necessary, the temperature effect can be compensated for simply by device calibration or by using external sensor data, while encapsulation^22^ of the sensor should eliminate the effects caused by the humidity variation of the environment.

## 4 Conclusions

In this work, printed piezoresistive sensors based on stretchable R2R manufactured PDMS-silver substrate–electrode surfaces and inkjet-deposited SWCNT micropatterns were studied. Through the optimization of surface wetting of the substrate and printing parameters of SWCNT inks, microscopic line and zigzag patterns of conductive networks of nanotubes with a sheet resistance of <100 Ω/□ could be deposited between stretchable electrodes on the plasma-treated substrate. The developed structures show high sensitivity to tensile strain (a gauge factor of up to 400), pressure (a sensitivity of ∼0.09 Pa^–1^), and convex bending deformation (a radius below 10 mm). As demonstrated, the printed stretchable structures were found to be suitable for mounting on the skin to monitor and resolve cardiac pulses measured at the radial artery, to monitor flexing of a finger, and to detect extension/contraction of chest upon inhaling/exhaling. Our study indicates that inkjet deposition of nanomaterials can complement well other printing technologies to produce flexible and stretchable sensors in a versatile manner and projects the development of a broad spectrum of devices to be implemented in applications related to soft robotics, medical equipment, sport apparel as well as in automotive, aeronautics, and civil engineering.

From the application point of view, a number of different parameters need to be optimized to ensure good sensor performance. For instance, Young’s modulus of the substrate should match the mechanical properties of the surface it is mounted on to enable good force/strain transfer between the two. In addition, the magnitude of strains and stresses during the deformation needs to be considered when selecting the thickness of the substrate and the size of the sensor. The device can be tuned for application by changing the dimensions and shape of the sensing material with respect to the total length of the stretching membrane, thus keeping the strain of the sensing component within the favorable region.^30^ As shown here and in the literature,^10−12,22^ meander, zigzag, or other Kirigami-like structures are useful to decrease the stress in the conductive patterns depending on the application. Furthermore, it was demonstrated that changes in humidity and temperature in the environment of the sensors can cause a slight drift of the base resistance of CNT films, which may be considered in the design.

## Supporting Information Available

Resolved X-ray photoelectron spectrum of Ar implanted in the substrate after plasma treatment; relative change of sensor device resistance when using a fast stage movement; table summarizing contemporary sensor devices based on carbon–polymer piezoresistive materials; and temperature/humidity effect on the relative change of the sensor device resistance (PDF)
