# Supplementary material for: Inkjet-Deposited Single-Wall Carbon Nanotube Micropatterns on Stretchable PDMS-Ag Substrate–Electrode Structures for Piezoresistive Strain Sensing
Source: ACS Appl Mater Interfaces. 2021 Jun 2;13(23):27284–94. doi: 10.1021/acsami.1c04397 (PMC8289179; doi:10.1021/acsami.1c04397)
Supplement: Supplementary file 1 — am1c04397_si_001.pdf [file am1c04397_si_001.pdf]

Supporting information

Inkjet deposited SWCNT micropatterns on stretchable PDMS-Ag substrate-electrode structure for piezoresistive strain sensing

Henri Ervasti,[1] *Topias Järvinen,*[1] *Olli Pitkänen,*[1] *Éva Bozó,*[1] *Johanna Hiitola-Keinänen,*[2] *Olli-Heikki Huttunen,*[2] *Jussi Hiltunen,*[2] *Krisztian Kordas*[1,*]

[1] Microelectronics Research Unit, University of Oulu, Erkki Koiso-Kanttilan katu 3, FIN-90570 Oulu, Finland

[2] VTT Technical Research Centre of Finland, Kaitoväylä 1, FIN-90590 Oulu, Finland

*Corresponding author

E-mail: krisztian.kordas@oulu.fi

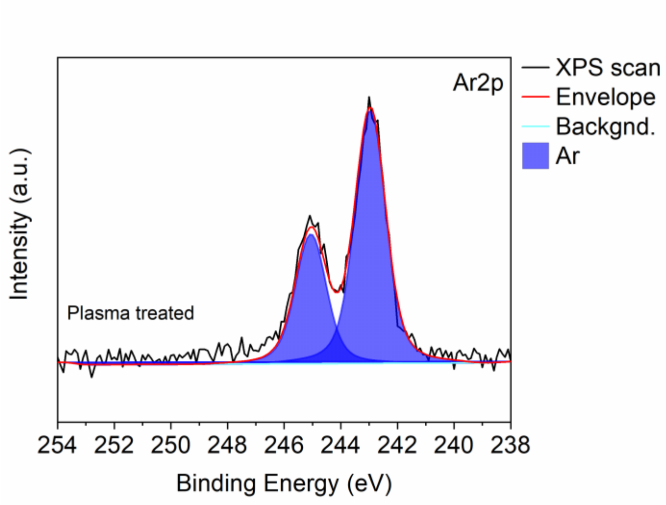

Figure S1. Resolved X-ray photoelectron spectra of Ar2p peaks after Ar plasma treatment of PDMS at 200 W for 13 min

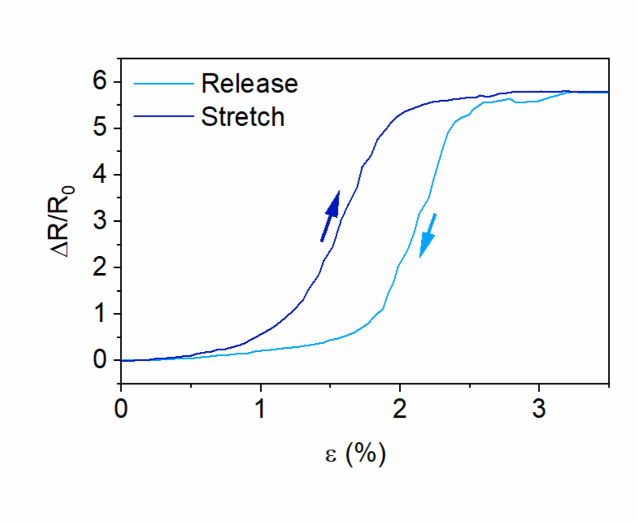

Figure S2. Relative change of the resistance of a sensor device with straight line using a high stage movement speed. [Figure is reproduced from Ref. 33 Ervasti, H., Inkjet-Printed SWCNT Conductors and Sensors on PDMS. M.Sc. thesis, 2020, University of Oulu.]

Table S1. Sensor properties of reported carbon based piezoresistive sensors in the literature

**Material**	Preparation	Gauge factor	Max strain	Demonstrated applications	Reference
**MWCNTs on EFTE**	Inkjet printing	1	0.25%	-	47
**MWCNT on PE**	Roll-to-roll print between PE	-	120%	Transparent touch sensor	48
**MWCNTs / graphite on rubber**	Deposition by hand	43.4 / 346.6	620% / 246%	-	49
**MWCNTs between PEN**	CNT transfer between PEN	-	-	Pressure, Joint movement	50
**MWCNT/PDMS**	Spin coating	70	50%	Joint and body movement	51
**Graphene on PDMS**	Transfer and etching	14	7.1%	Joint movement	52
**Graphene on PMMA**	Transfer and etching	4.33	0.5%	-	53
**Graphene on PDMS**	Transfer, etching and RIE	600	1%	Joint movement	54
**Graphene on PDMS**	Transfer, and etching	223	3%	Joint movement	55
**Graphene foam**	Freeze casting	1.3	60%	Breath monitoring	56
**Graphene on rubber**	Molding	164.5	12%	Joint and body movement, vibration	57
**rGO coated fiber**	Dip coating	8.8	110%	Joint movement	58
**rGO/DIW liquid in Ecoflex**	Casting???	31.6	400%	Joint and body movement	59
**rGO-PDMS ink on SU-8**	Direct ink writing	20.3	40%	Tactile sensor, Joint and body movement	60
**Carbonized crepe paper with cellulose**	Encapsulated in PDMS	10.1	5%	Body joint movement, Robot arm	61
**SWCNTs on PDMS**	Inkjet printing (Straight pattern)	400	2.5%	Joint and body movement, pulse	This work
**SWCNTs on PDMS**	Inkjet printing (Zigzag pattern)	28	2.5%	Joint and body movement, pulse	This work

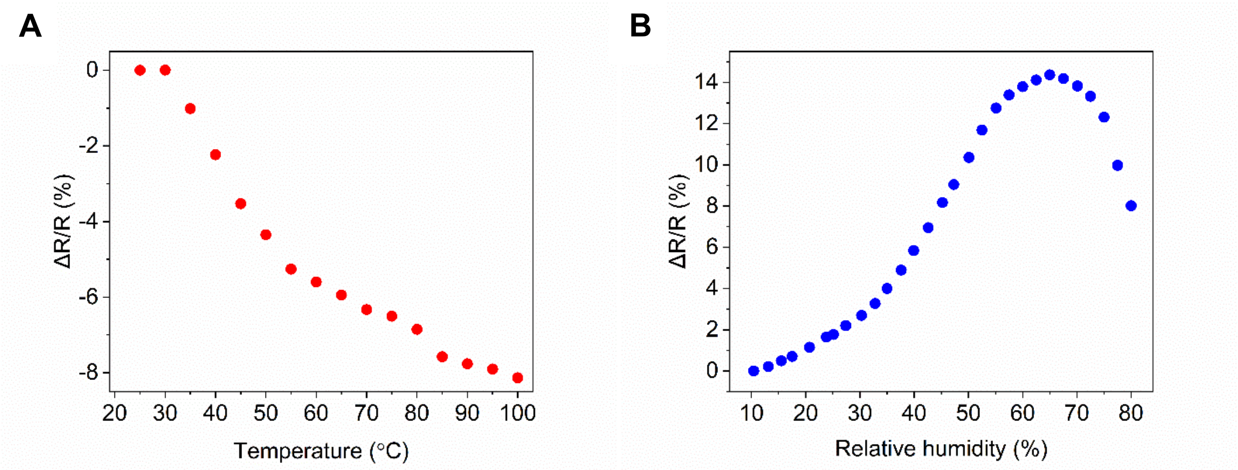

Figure S3. Relative change of the resistance of a sensor device (straight line pattern) as a function of **(A)** temperature (in synthetic air flow) and **(B)** relative humidity (at 25 °C).
